# Special Issue: “Viral Replication Complexes”

**DOI:** 10.3390/v13101902

**Published:** 2021-09-23

**Authors:** Núria Verdaguer, Diego S. Ferrero

**Affiliations:** Institut de Biología Molecular de Barcelona, CSIC, Parc Científic de Barcelona, Baldiri Reixac 15, 08028 Barcelona, Spain

Viruses are extraordinary biological entities that can only thrive as obligate intracellular parasites, exploiting other living cellular components in order to reproduce. Despite the diversity in the virosphere, a central component of the viral replication machinery is the nucleic acid polymerases that are responsible for copying the viral genome and for generating genetic diversity. In the RNA virus world, with the exception of retroviruses, the RNA-dependent RNA polymerases (RdRPs) catalyze RNA synthesis, functioning either as single polypeptides, or in a complex with other viral or host components to transcribe and replicate viral RNA genomes. In this Special Issue, we seek to highlight recent progress in our understanding of the structure and function of different viral replication complexes. Four original studies and seven reviews contributed to the issue to increase our general knowledge of RNA virus replication.

From the original articles, Ferrero and colleagues describe a remarkable series of X-ray structures of the non-canonical RdRP of the (+) ssRNA Thosea asigna virus in a complex with RNA, nucleotide substrates and the ions involved in its catalytic activity [1]. The binary and ternary complexes obtained allowed us to understand the atomic interactions governing different events of the RNA polymerization process, such as nucleotide addition, ion binding and RNA elongation, highlighting important mechanistic similarities between non-canonical and canonical RdRPs.

Albentosa-González and colleagues explain the effects of phosphorylation on the modulation of RdRP activity in the Usutu, Zika and West Nile viruses (USUV, ZIKV, WNV) [2]. The flavivirus non-structural protein 5 (NS5) comprises an N-terminal S-adenosyl-L-methionine (SAM)-dependent methyltransferase (MTase) domain and the RdRP domain at the C-terminus. The phosphorylation of the RdRP domain by Akt kinase compromises the primer extension activity in USUV and ZIKV, but not in WNV.

Song and colleagues provide new insights into therapeutic intervention against AIDS, using viral antigen-specific iPSCs to suppress HIV replication in a murine model [3].

Finally, we dive into the conformational dynamics of the poliovirus multifunctional 3CD polyprotein, guided by the work of Winston and Boehr [4]. The picornaviral 3CD protein is the precursor of the 3C protease and 3D RdRP but has different protease specificity than the processed 3C. It is inactive as RdRP and participates in multiple protein–protein, protein–RNA and protein–lipid interactions during viral replication. The authors of this article performed a complete NMR spectroscopy study to identify the differences in the conformational dynamics of functionally important regions that are poorly described by the available crystal structures.

The reviews in this Special Issue leave no doubt that exciting new discoveries in the regulation of genome replication in (+) and (−) ssRNA viruses are far from over. The seven reviews in this section cover a broad range of topics. Domingo and colleagues consider the error rates displayed by the RdRPs during template copying as an important parameter to understand the genetic and antigenic variability of RNA viruses. However, there is a limit to the number of mutations that can be accepted to preserve de-encoded genetic information. Authors also review how, in some viruses (e.g., coronaviruses), mutation rates may be modulated by proofreading repair activity [5]. This Special Issue also provides up-to-date knowledge about the Flavivirus replication process. Morita and Suzuki review the formation and function of the membrane-associated flaviviral replication organelle and its regulation by cellular factors [6]. Van den Elsen and colleagues discuss the molecular structures of the nonstructural proteins and their interactions with each other and RNA, as well as the ultrastructural characterization of the replication complex by electron microscopy and tomography [7]. In addition, a model of how NS5 polymerases manage to recognize the regulatory stem loop A at the 5′ terminus of the viral genome to initiate RNA synthesis at the 3′ terminus is presented in the review of Choi [8]. Finally, Li and colleagues summarize the current knowledge on viral and cellular factors involved in the regulation of the hepatitis C virus (HCV) genome replication and the development of direct-acting antivirals against HCV replication [9].

For the (−) ssRNA virus world, the review by Cao and colleagues updates the molecular insights into the replicative complex of the respiratory syncytial virus (RSV), reviewing the fundamentals of RNA synthesis and proposing a model of multiprotein coordination during different RNA synthesis processes [10]. In addition, Weis and te Vethuis summarize the interplay between the RNA synthesis of influenza virus and the detection of influenza virus RNA via the innate immune system [11].

Finally, we would like to acknowledge all the authors for their contributions to this Special Issue. It has been a pleasure to read their work and learn from it. We will encourage our colleagues to continue with their exceptional research, even during the difficult times of the SARS-CoV-2 pandemic. Because of this pandemic, we are more conscious of the transcendence of understanding viral replication and the multiple mechanisms that viruses use to regulate this essential activity during infection.
